# Type 1 Conventional CD103^+^ Dendritic Cells Control Effector CD8^+^ T Cell Migration, Survival, and Memory Responses During Influenza Infection

**DOI:** 10.3389/fimmu.2018.03043

**Published:** 2018-12-21

**Authors:** See Liang Ng, Yi Juan Teo, Yolanda Aphrilia Setiagani, Klaus Karjalainen, Christiane Ruedl

**Affiliations:** School of Biological Sciences, Nanyang Technological University, Singapore, Singapore

**Keywords:** influenza, dendritic cell, CD103, CD8^+^ T cell, migration, survival, inflammation, Clec9A

## Abstract

Type 1 conventional CD103^+^ dendritic cells (cDC1) contribute significantly to the cytotoxic T lymphocyte (CTL) response during influenza virus infection; however, the mechanisms by which cDC1s promote CTL recruitment and viral clearance are unclear. We demonstrate that cDC1 ablation leads to a deficient influenza-specific primary CD8^+^ T cell response alongside severe pulmonary inflammation, intensifying susceptibility to infection. The diminished pulmonary CTL population is not only a consequence of reduced priming in the lymph node (LN), but also of dysregulated CD8^+^ T cell egression from the LN and reduced CD8^+^ T cell viability in the lungs. cDC1s promote S1PR expression on CTLs, a key chemokine receptor facilitating CTL LN egress, and express high levels of the T cell survival cytokine, IL-15, to support CTL viability at the site of infection. Moreover, cDC1 ablation leads to severe impairment of CD8^+^ T cell memory recall and cross-reactive protection, suggesting that cDC1 are not only involved in primary T cell activation, but also in supporting the development of effective memory CD8^+^ T cell precursors. Our findings demonstrate a previously unappreciated and multifaceted role of CD103^+^ DCs in controlling pulmonary T cell-mediated immune responses.

## Introduction

Pulmonary conventional dendritic cells (cDCs) are pivotal for the initiation of immune responses upon pathogen intrusion via the intranasal route. Two major cDC subsets, CD103^+^ migratory cDCs (cDC1) and CD11b^+^ migratory cDCs (cDC2), are known to act as key mediators of the cytotoxic T lymphocyte (CTL) response against influenza A virus (IAV) infection. Mounting evidence indicates that these DC subpopulations regulate distinct aspects of the primary CTL response. In particular, the absence of CD103^+^ migratory cDCs has been shown to significantly increase disease mortality (1, 2).

CTLs are essential for host resistance against IAV infection. To generate an effective CTL response, migratory cDCs must acquire virus antigen from the dying, infected epithelial cells, and migrate to the lung-draining mediastinal lymph node (mLN) where they subsequently cross-present to antigen-cognate naïve CD8^+^ T cells (3). These virus-specific CD8^+^ T cells undergo extensive expansion *in situ* in the LN and travel back to the infected lung where they recognize and eliminate virus-infected cells. The magnitude of the virus-specific CTL population in the lung directly determines the host resistance, thus mechanisms regulating CTL numbers are central to host countermeasures (4, 5). Ablation of CD103^+^ cDC1s in Langerin-DTR and Batf3^−/−^ transgenic mice has been shown to significantly diminish the virus-specific CTL population in models of mouse infection (1, 6), although the specific mechanisms regulating virus-specific CTL numbers in the respiratory tract, as well as the development of memory CD8^+^ T cell responses, have not been fully elucidated.

Here, we demonstrate that CD103^+^ cDC1s regulate virus-specific CD8^+^ T cell trafficking, and directly promote CTL survival in the lung. We further show that activation of antigen-cognate naïve CD8^+^ T cells in the mLN is predominantly coordinated by CD103^+^ migratory cDC1s, with little contribution from either CD11b^+^ migratory cDC2s or LN-resident cDCs. Moreover, while the induction of neutralizing antibodies against virus surface proteins is unaltered by the absence of CD103^+^ cDC1s, there is a clear defect in the memory CD8^+^ T cell-mediated recall response under these conditions. These multifaceted properties position cDC1s as central regulators of the host immune response to IAV.

## Materials and Methods

### Mouse Strains

Clec9A-DTR transgenic mice were generated in our laboratory via a BAC recombineering approach in a BALB/c genetic background (7), and subsequently cross bred with C57BL/6 for 10 generations. Clec9A-DTR C57BL/6 transgenic mice, together with wild type C57BL/6, were bred and maintained under specific pathogen-free (SPF) conditions in the Nanyang Technological University (NTU) animal facility. All experiments were approved by the Institutional Animal Care and Use Committee under the number ARF- SBS/NIE A-0375AZ.

### Influenza Virus Infection

Influenza virus strain A/PR/8/34, PR8 (H1N1), and recombinant virus OVA-PR8 were gifts from Dr. Sivasankar Balasubramanian (6). Influenza virus strain A/X-31 (H3N2) was a gift from Prof. David Michael Kemeny. PR8 virus was used in all influenza experiments. X-31 virus was used to immunize mice prior to secondary lethal PR8 challenge in the heterosubtypic immunity experiment. Each mouse was anesthetized (ketamine, 10 mg/kg body weight, and xylazine, 2 mg/kg body weight) before intranasal delivery of PR8/X-31 virus prepared in 30 μl of PBS. Female mice (6–8 weeks of age) were used for influenza infections.

### Diphtheria Toxin-Mediated DC Ablation

Diphtheria toxin (DT; 20 ng/ gram body weight) was prepared in PBS supplemented with 1% mouse serum. For DC ablation profiling, Clec9A-DTR mice were administered intraperitoneally (i.p.) two consecutive doses of DT and were sacrificed 24 h after the second dose of DT. For Clec9A-DTR mice infected with influenza virus, two DT doses were given prior to infection, after which Clec9A-DTR mice were given DT once every 3 days until experimental completion. For homosubtypic and heterosubtypic infection experiments, two DT doses were given to Clec9A-DTR mice prior to infection and DT administration (once every 3 days) continued for the following 2 weeks. No DT was administered during secondary challenge.

### Tissue Collection, Processing, and Cell Isolation (8)

Broncho-alveolar lavage (BAL) fluid was extracted by performing lung lavage three times, each with 0.5 ml PBS, to retrieve cells that reside in the alveolar compartments. After BAL extraction, lung tissues were perfused with 10 ml PBS before excision. Excised lung tissues were minced and incubated in IMDM supplemented with 2 mg/ml collagenase D (Life Technologies, Carlsbad, CA, USA) for 60 min at 37°C. Subsequently, lung tissues were meshed and passed through a 70-μm cell strainer to obtain single-cell suspensions. The cell suspensions were resuspended in 5 ml of 35% Percoll^TM^ (GE Healthcare Life Science, Chicago, IL, USA) before centrifuging at 600 × g for 10 min at room temperature (RT). After RBC lysis cells were resuspended in PBS supplemented with 2% bovine serum (PBS 2%). For the processing of mLNs, dissected mLNs were minced and incubated in 2 mg/ml collagenase D for 60 min. For cell counting, small aliquots of BAL, lung, and mLN single-cell suspensions were premixed with Trypan blue prior to counting on a hemocytometer.

### Cell Labeling for Flow Cytometry Analyses

For staining of cell surface antigens, single-cell suspensions from BAL, lung, and mLN were incubated with fluorochrome-labeled antibodies at 4°C for 20 min, washed, and resuspended in PBS 2% for analysis. Ki-67 intracellular staining (1:350) was performed according to the manufacturer's instructions (eBioscience, San Diego, CA, USA). For detection of intracellular cytokines, lung single-cell suspensions were incubated in IMDM medium with or without PMA/Ionomycin (PMA 10 ng/ml, Ionomycin 1 μg/ml, Sigma-Aldrich, St. Louis, MO, USA) for 6 h and Brefeldin A (10 μg/ml) was added for the final 3 h. Cells were stained for cell surface antigens, fixed, and permeabilized before staining with anti-IFN-γ and anti-IL-10 antibodies (1:500, BioLegend, San Diego, CA, USA) prepared in 0.05% saponin. Stained cells were subsequently washed and resuspended in PBS 2% for analysis.

To stain for NP_366−374_-specific CD8^+^ T cells, PE-labeled H-2D^b^ MHC class I Dextramer^TM^ of Influenza A Nucleoprotein epitope ASNENMETM (NP_366−374_) (Immudex, Copenhagen, Denmark) was used. Briefly, single-cell suspensions from BALs, lungs, and mLNs were stained with Live/Dead Fixable ® Violet Dead Cell stain following the manufacturer's instructions (Molecular Probes Thermo Fisher Scientific, Singapore), after which the cells were stained with PE-labeled H-2K^b^ MHC Dextramer^TM^ NP_366−374_. For detection of cell death and apoptosis, lung single-cell suspensions were stained for Annexin V followed by staining for H-2K^b^ MHC Dextramer^TM^ NP_366−374_ and other cell surface antigens. Stained cells were subsequently washed with PBS 2% and resuspended in PBS 2% containing propidium iodide (PI) for flow cytometry analysis.

Samples were acquired on a BD LSR Fortessa flow cytometer (BD Biosciences, San Jose, CA, USA). Leukocytes were gated based on forward- and side-scatter properties (FSC, SSC), and live cells were gated based on exclusion of cells staining positive for PI or Live/Dead Fixable® Violet Dead Cell stain.

### *In vitro* T Cell Proliferation Assay

Wild type mice mLN and spleen were harvested 5 days after PR8 infection and were sorted for individual migratory DC and resident DC subsets (Supplementary Figure S3). Sorted DCs were incubated in 96-well round-bottom plates (Corning, Corning, NY) with purified naïve CFSE-labeled OT1-TCR CD8^+^ T cells (1:20 ratio) in IMDM supplemented with 7% FCS. After 4–5 days of incubation at 37°C, the cells were labeled with antibodies and then analyzed for CFSE dilution by flow cytometry.

### *In vivo* T Cell Proliferation Assay

Naïve CD8^+^ T cells were purified from spleens of *Rag1*^−/−^OT1-TCR mice by LS column positive selection (Miltenyi Biotec, Bergisch Gladbach, Germany) according to manufacturer's instructions. Purified CD8^+^ T cells were labeled with 10 μM CFSE for 11 min at 37°C water bath and 1 ml of 100% FCS was added immediately after. Labeled cells were washed with 10 ml of PBS for 3 times. A total of 2 × 10^6^ CFSE-labeled CD8^+^ T cells were injected i.v. to individual wild type or Clec9A-DTR recipients. Recipient mice were infected with OVA-PR8 virus 3 h prior the adoptive T cells transfer.

S1PR surface staining was done using anti-mouse S1P_1_ biotinylated antibody clone #713412 (R&D systems, Minnesota, United States) followed by streptavidin- BUV395 incubation.

### Preparation of Bone Marrow-Derived CD103^+^ DCs (iCD103^+^ DCs) (9)

Bone marrow (BM) cells were extracted by flushing the femur and tibia bone cavity repeatedly with PBS 2%. The cells were centrifuged and resuspended in RBC lysis buffer for 10 min at RT. Cells were resuspended in IMDM 2% supplemented with GM-CSF (5 ng/ml) and Flt3L (200 ng/ml) at density of 10^7^ cells/ml and incubated at 37°C. After 6–7 days, non-adherent cells were collected and resuspended with fresh GM-CSF and Flt3L-supplemented IMDM 2% at a density of 3–5 × 10^6^ cells/ml. After 8 days, cells were stained and sorted for iCD103^+^ DCs. Purified iCD103^+^ DCs (5 × 10^6^/mouse) were administered via the intranasal route.

### Quantitative Real-Time PCR

Lung tissue was harvested and immediately homogenized in TRIzol^TM^ reagent (Thermo Fisher Scientific). Total RNA was subsequently purified using the RNAsimple Total RNA kit (Tiangen Biotech Ltd, Beijing, China). Real-time PCR was performed according to the manufacturer's instructions using the Primer design Precision® FAST protocol (Primerdesign Ltd, Cambridge, UK). Primer sequences were as follows: *Virus M1*; Fwd: GGACTGCAGCGTTAGACGCTT, Rev: CATCCTGTTGTATATGAGGCCCAT, *IFN-* γ; Fwd: CACGGCACAGTCATTGAAAG, Rev: CCAGTTCCTCCAGATATCCAAG, *IL-6*; Fwd: CTCTGGGAAATCGTGGAAAT, Rev: CCAGTTTGGTAGCATCCATC, *IL-12p40*; Fwd: CTAGACAAGGGCATGCTGGT, Rev: GAAGCAGGATGCAGAGCTTC, *IL-10*; Fwd: CAGAGCCACATGCTCCTAGA, Rev: TGTCCAGCTGGTCCTTTGTT, *TNF-* α; Fwd: TCTTCTCATTCCTGCTTGTGG, Rev: GGTCTGGGCCATAGAACTGA, *GM-CSF*; Fwd: GCATGTAGAGGCCATCAAAGA, Rev: CGGGTCTGCACACATGTTA, *M-CSF*; Fwd: GGTGGAACTGCCAGTATAGAAAG, Rev: TCCCATATGTCTCCTTCCATAAA, *IL-15*; Fwd: CATCCATCTCGTGCTACTTGTGTT, Rev: CATCTATCCAGTTGGCCTCTGTTT and β *-Actin*; Fwd: AAGGCCAACCGTGAAAAGAT, Rev: CCTGTGGTACGACCAGAGGCATACA.

### *In vivo* Assay of Blood Vessel Permeability—Evans Blue dye

Mice were administered i.v. with Evans blue dye 30 min before harvest. Lungs and spleens were extracted and dabbed dry before transferring to 1.5 ml Eppendorf tubes containing 500 μl of formamide to extract Evans blue dye from tissues. Tubes were incubated for 36–48 h at 55°C before absorbance measurement at OD_610_. Measurements were tabulated as absorbance per milligram of tissue.

### Cytokine ELISA

BALs were collected from mice infected for 6 and 10 days. ELISAs for IFN-γ, IL-6, TNF-α, GM-CSF, IL-10, and IL12-p40 cytokines were conducted according to the manufacturer's instructions (eBioscience).

### Serum Passive Immunization

Clec9A-DTR and wild type mice were infected with 8 PFU influenza virus PR8. After 10 days of infection, retro-orbital bleeding was conducted on all infected mice. Collected blood was centrifuged at 11,000 × g for 10 min at 4°C, and the upper layer serum solution was collected. Subsequently, 50 μl serum was transferred i.v. to naïve mice 1 day prior to intranasal challenge with 32 PFU or 64 PFU influenza virus PR8.

### Histological Analysis

Lungs were dissected from wild type and Clec9A-DTR mice infected for 10 days. Dissected tissue was fixed in 4% phosphate-buffered paraformaldehyde for 48 h, dehydrated, and embedded in paraffin. Six-micron sections were stained with H&E and analyzed by bright field light microscopy at 80 × magnification. Images were obtained using a Nikon Eclipse 80i microscope.

### Computer Software

Flow cytometry data were analyzed using FlowJo 7.6.1 software (Tree Star, Inc., Ashland, OR). Graphs and statistical analyses were generated using Graphpad Prism 5.0 software (GraphPad Software, La Jolla, CA, USA).

### Statistical Analyses

Survival curves were analyzed using the Mantel-Cox long-rank test. Statistical significance between two groups was analyzed using the unpaired student *t*-test, and, for more than two groups, using a one-way ANOVA followed by Bonferroni test. Two-way ANOVA with Bonferroni test was used for data comprising more than two groups and two time-points. Statistical significance is demonstrated in the figures with asterisks: ^*^p < 0.05, ^**^p < 0.01, ^***^p < 0.001. GraphPad Prism 5.0 was used to analyze the data.

## Results

### Clec9A-DTR Mice Succumb to a Sub-lethal Influenza Infection

**Figure 1:**
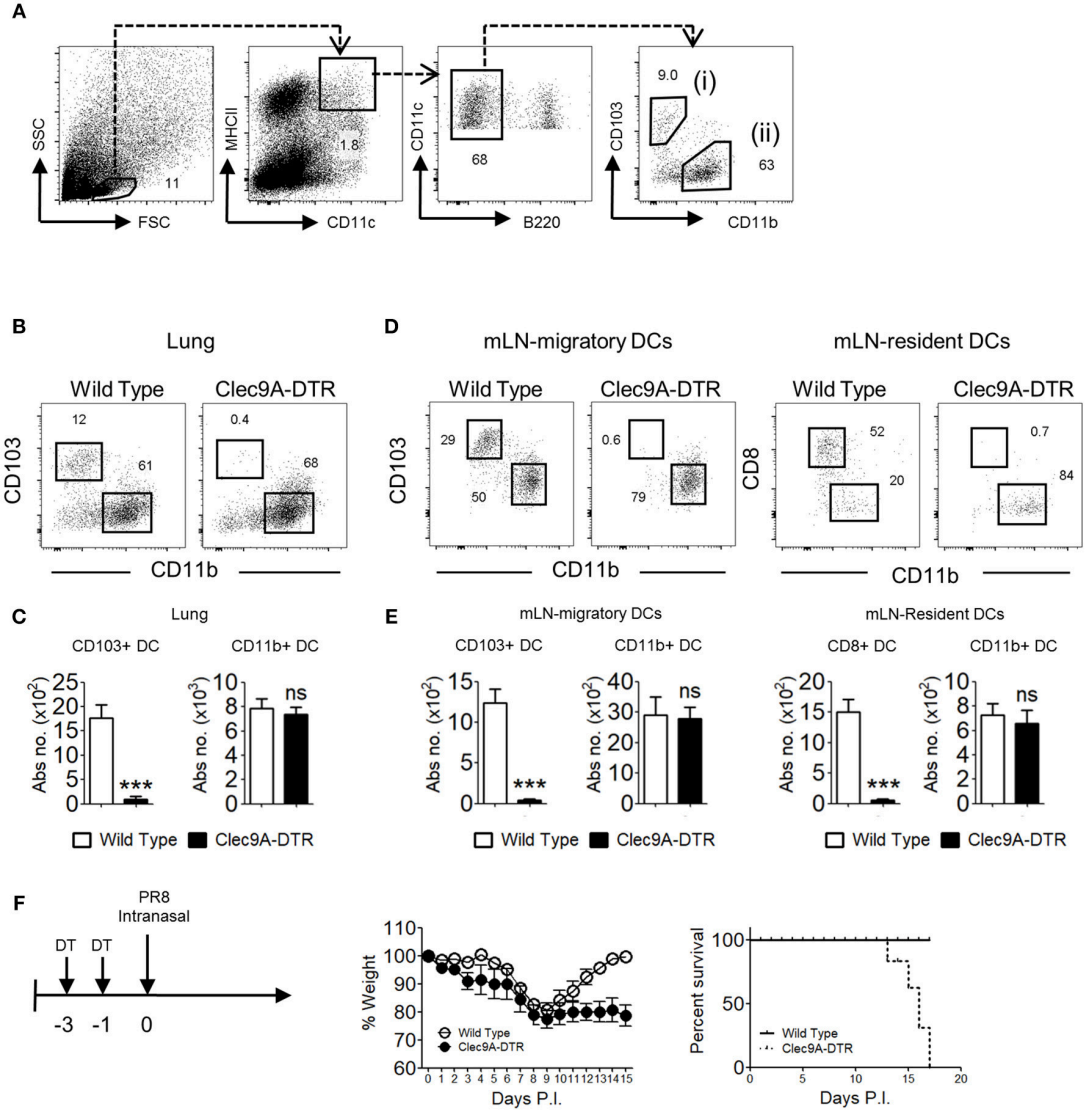
Clec9A-DTR mice succumb to a sub-lethal influenza infection. **(A)** Gating strategy for migratory CD103^+^ cDC1 in the lung, defined as MHCII^hi^CD11c^hi^B220^−^CD11b^−^CD103^+^ **(B)** Representative ablation profile of migratory CD103^+^ cDC1s in the lung of Clec9A-DTR mice after 2 consecutive days of DT administration. **(C)** Absolute numbers of migratory cDCs in the lung of DT-treated wild type and Clec9A-DTR mice. **(D)** Representative ablation profile of migratory CD103^+^ cDC1s and resident CD8^+^ cDC1s in the mLN of Clec9A-DTR mice after two consecutive days of DT administration. **(E)** Absolute numbers of migratory and resident cDCs in the mLN of DT-treated wild type and Clec9A-DTR mice. **(F)** Representative weight loss (left) and survival (right) curve of wild type and Clec9A-DTR mice after 16 PFU of PR8-H1N1 infection. Data are shown as mean ± SEM. ^***^p < 0.001. Data represent two **(C,E)** (*n* = 3–5) or three **(F)** (*n* = 4) independent experiments.

Migratory DCs are crucial antigen presenting cells (APCs) that deliver pathogen-derived antigen to cognate T cells in the draining LNs. Two distinct CD11c^hi^MHCII^+^ DC populations are found in the lungs, which can be discriminated by differential expression of CD103 (cDC1) and CD11b (cDC2) (Figure 1A). Similar populations are found in the mLN, together with two major resident DC subpopulations expressing CD8^+^ and CD11b^+^ (Supplementary Figure S1). Migratory CD103^+^ cDC1s express several unique markers, including the C-type lectin receptor, Clec9A. The Clec9A-DTR mouse is therefore a valuable animal model to assess the function of this particular DC subset (7, 10, 11). Because Clec9A is specifically expressed on cDC1, only CD103^+^ cDC1s, and not CD11b^+^ cDC2s are ablated in the lungs of Clec9A-DTR mice upon DT administration (Figures 1B,C). In the mLN, both migratory CD103^+^ cDC1s and resident CD8^+^ cDC1s are ablated, whereas the migratory CD11b^+^ cDC2 and resident CD11b^+^ cDC2 populations remained unaffected in the DT-treated Clec9A-DTR mouse (Figures 1D,E).

Studies utilizing alternative CD103^+^ cDC1 ablation mouse models, such as the Langerin-DTR transgenic mouse, have demonstrated the importance of CD103^+^ cDC1s in influenza resistance (1, 6). Here we used the Clec9A-DTR mouse model to investigate the specific mechanisms underlying the protective effect mediated by cDC1. First, to confirm the contribution of CD103^+^ cDC1 in anti-influenza immunity, we infected wild type and Clec9A-DTR mice with PR8 virus and monitored their weight loss. After an initial 20% body weight loss, infected wild type mice survived the infection and recovered completely within the first 2 weeks. In contrast, Clec9A-DTR mice were unable to recover from a similar initial weight loss and all mice succumbed to IAV infection by 2 weeks (Figure 1F). Hence, our results recapitulate previous findings that CD103^+^ cDC1s contribute to protection against primary IAV infection (1, 6).

### Impaired Effector CD8^+^ T Cell Response in the Absence of Pulmonary CD103^+^ cDC1s

**Figure 2:**
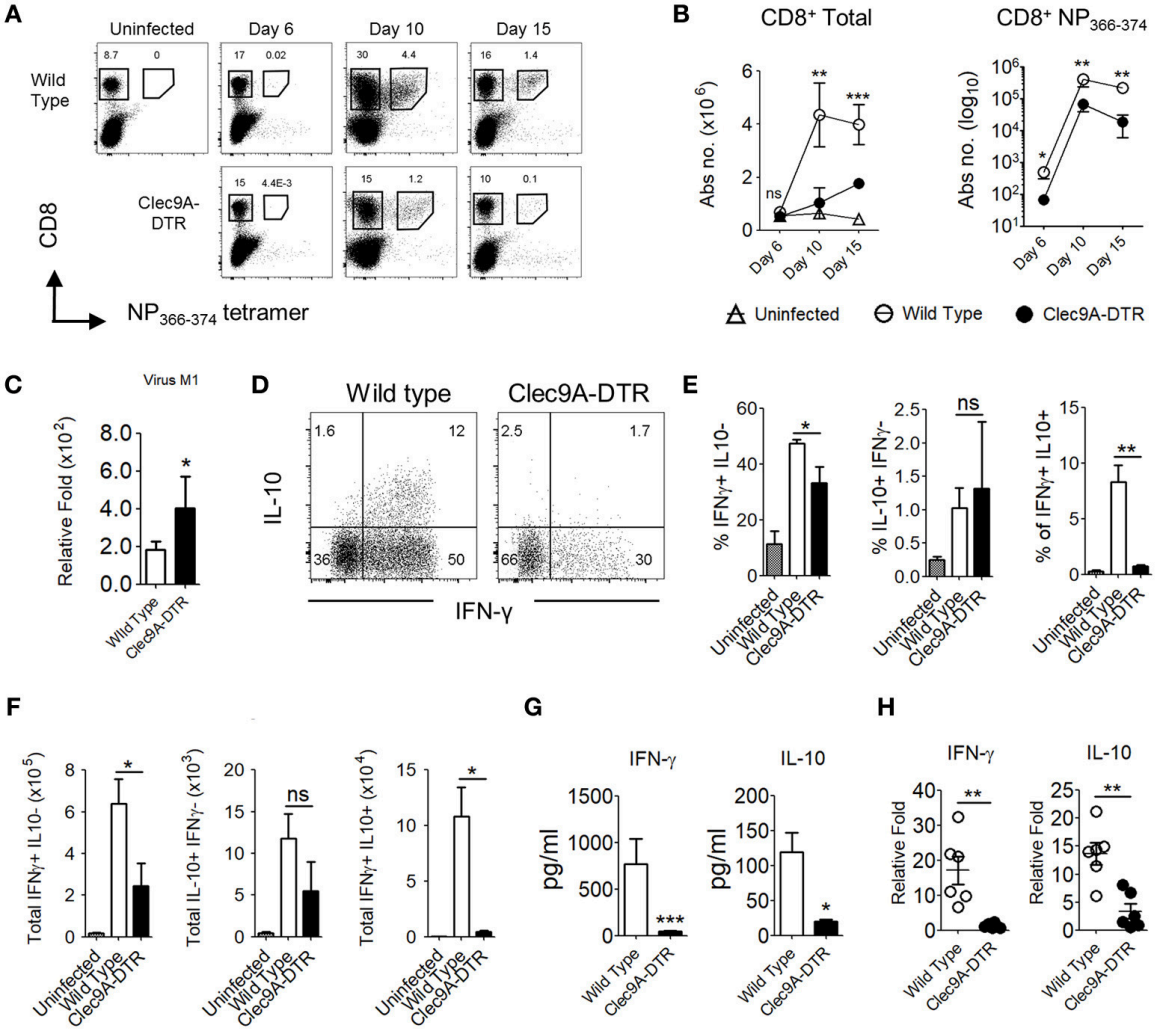
Impaired effector CD8^+^ T cell responses in the absence of pulmonary CD103^+^ cDC1s. **(A)** NP_366−374_-specific CD8^+^ T cells in the lungs of uninfected, infected wild type, and Clec9A-DTR mice (day 6, 10, and 15 post infection). **(B)** Kinetics of total CD8^+^ T cells and NP_366−374_-specific CD8^+^ T cells in the lungs of uninfected, infected wild type, and Clec9A-DTR mice. Absolute numbers are shown. **(C)** Lung virus load was measured by relative quantification of M1 viral protein in infected wild type and Clec9A-DTR mice on day 10 post infection. **(D–F)** Lung cells were harvested from uninfected, infected wild type, and Clec9A-DTR mice on day 10 post infection and stimulated with PMA/Ionomycin for 3 h followed by Brefeldin A incubation for an additional 3 h. Intracellular IFN-γ and IL-10 staining profiles **(d)** of pulmonary CD8^+^ T cells, frequency **(E)** and total numbers **(F)** of IFN-γ-producing, IL-10-producing, and IFN-γ/IL-10 double-producing CD8^+^ T cells in the lungs. **(G)** IFN-γ and IL-10 BAL levels as measured by sandwich ELISA **(H)** Relative quantification of IFN-γ and IL-10 transcripts. Data are shown as mean ± SEM. ^*^p < 0.05. ^**^p < 0.01. ^***^p < 0.001. Data represent two **(B–F)** (*n* = 3–5) independent experiments.

CD103^+^ cDC1s are highly effective cross-presenting cells (12, 13); therefore, it is unsurprising that in the absence of CD103^+^ cDC1s, numbers of total and NP_366−374_-specific CD8^+^ T cells were significantly reduced (Figures 2A,B). Accordingly, higher levels of IAV load were detected in the lungs of infected Clec9A-DTR mice compared with wild type mice (Figure 2C). Consistent with the observed decrease in effector CTL population, the frequency of IFN-γ and IL-10 secreting cells were lower in Clec9A-DTR mice (Figure 2D), in particular, IL-10 secreting cells were distinctively sparse (Figure 2D). Consistent with the observed decrease in effector CTLs, the frequency and numbers of IFN-γ and IL-10 secreting cells were lower in Clec9A-DTR mice (Figures 2D–F), in particular, IL-10 producing cells were distinctively sparse (Figure 2F). Correspondingly, reduced levels of IFN-γ and IL-10 were detected in BAL from Clec9A-DTR mice (Figures 2G,H) (14). On the contrary, naïve CD4^+^ T cells in the mLN can be efficiently activated by both CD103^+^ cDC1s and CD11b^+^ cDC2s (15), the redundant role of CD103^+^ cDC1s was confirmed by the unaffected CD4^+^ T cells cytokine response in the mLN obtained from Clec9A-DTR mice (Supplementary Figures S2B,C). On the other hand, CD103^+^ cDC1s ablation did considerably reduce the CD4^+^ T cells cytokine response in the lung (Supplementary Figures S2B,D).

### Enhanced Pulmonary Inflammation and Severe Lung Damage in IAV Infected Clec9A-DTR Mice

**Figure 3:**
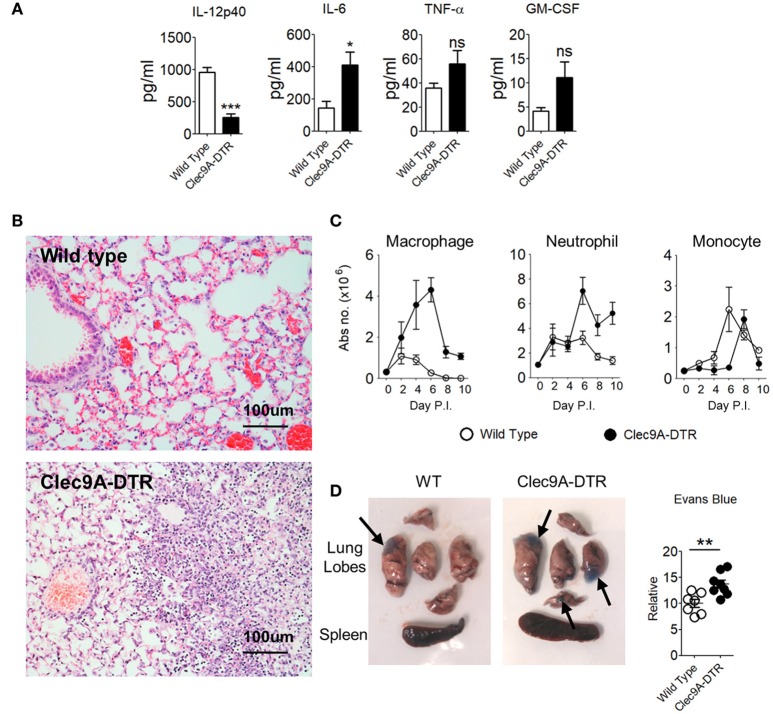
Enhanced pulmonary inflammation and severe lung damage in IAV-infected Clec9A-DTR mice. **(A)** IL-12p40, IL-6, TNF-α, and GM-CSF BAL levels measured by sandwich ELISA (day 10 post infection). **(B)** Representative H&E histology images of lungs from day 10-infected wild type and Clec9A-DTR mice. Magnification 80×. **(C)** Total number of macrophages, neutrophils, and monocytes in the lungs on day 2, 4, 6, 8, and 10 post infection. **(D)** Images of lungs from day 10-infected wild type and Clec9A-DTR mice 30 min after administration of Evans blue dye (left and middle). Relative quantification calculated from absorbance readings at OD_610_ (right). Black arrows indicate Evans blue dense regions. Data are shown as mean ± SEM. ^*^p < 0.05. ^**^p < 0.01. ^***^p < 0.001. Data represent two **(A–D)** (*n* = 3–5) independent experiments.

Elevated cytokine levels accompanying massive infiltration of innate immune cells is a hallmark of the early phase of IAV infection (16). In sub-lethal infections, the innate response is able to stifle viral replication temporarily, but complete viral elimination requires an adaptive T cell response. Incomplete viral clearance from the lungs results in a continuous and persistent inflammatory cytokine milieu. Consistent with a high viral load (Figure 2A), proinflammatory IL-6 levels were significantly higher in BAL fluid from Clec9A-DTR mice (Figure 3A) compared with infected WT controls. TNF-α and GM-CSF were also slightly elevated in the absence of cDC1s, although this was not significant at the protein level (Figure 3A and Supplementary Figure S2A). As expected, IL-12 levels were lower in Clec9A-DTR mice given that a substantial amount of IL-12 is produced by CD103^+^ cDC1s (Figure 3A), a reduction which significantly impacted the IFN-γ response (Figures 2D–F).

Histological analyses indicated increased edema, eosinophilic depositions, hemorrhages, and cellular infiltrations in the lungs of Clec9A-DTR mice (Figure 3B). Among the infiltrating cell population, neutrophils and macrophages, but not monocytes, accounted for the majority of infiltrates (Figure 3C). Higher Evans blue dye retention was also apparent in Clec9A-DTR lungs (Figure 3D), indicating altered vasculature permeability. Taken together, the absence of cDC1s enhances local pulmonary inflammation as a result of massive inflammatory cell infiltration, resulting in severe lung pathology.

### CD103^+^, but Not CD8^+^ cDC1s, Cross-Present Virus Antigen in the mLN

**Figure 4:**
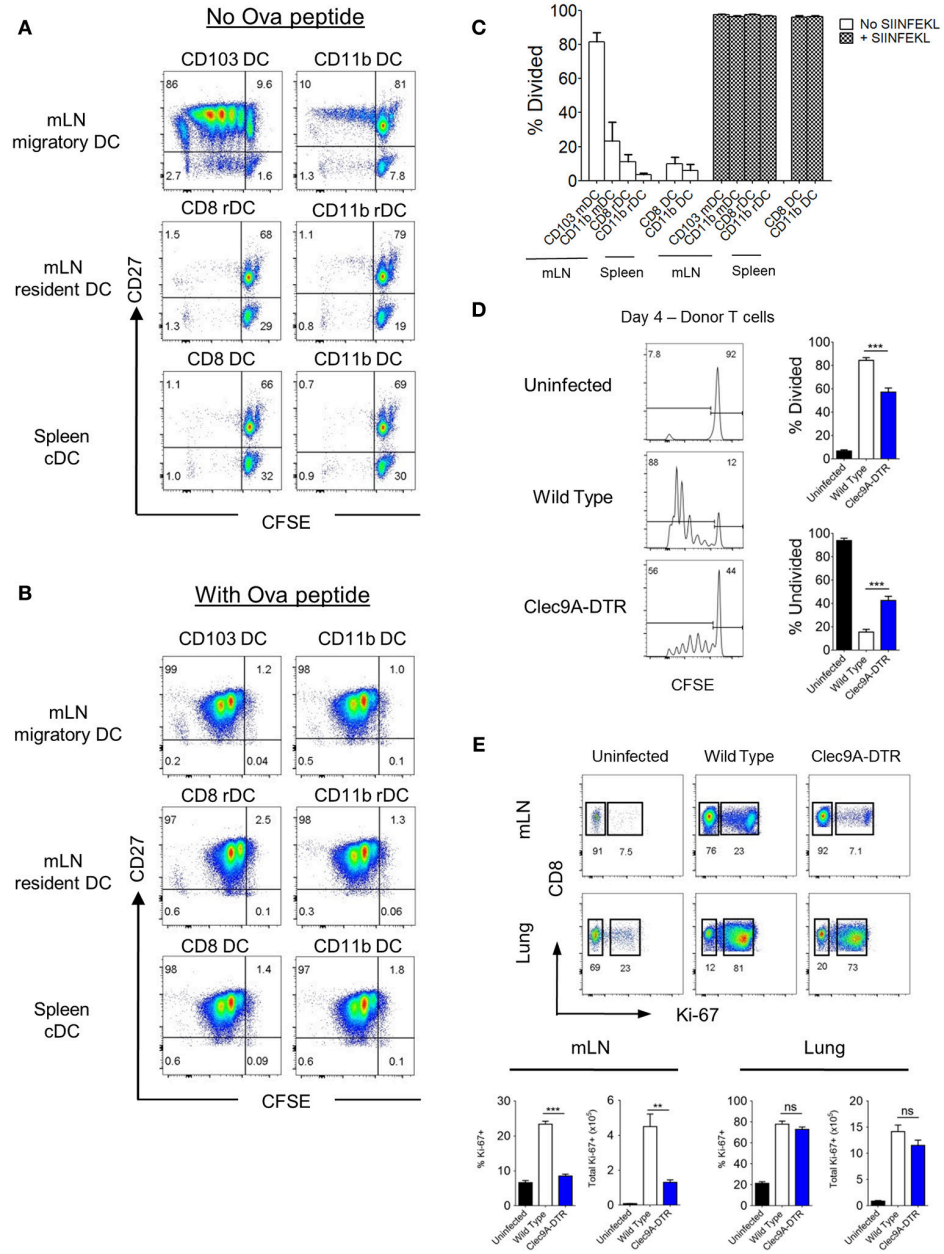
CD103^+^, but not CD8^+^ cDC1s, cross -present virus antigen in the mLN. **(A,B)** Spleen and mLN DCs were sorted from wild type mice infected with recombinant OT1-PR8 virus for 5 days. Sorted DCs were individually co-cultured with CFSE-labeled OT1-CD8^+^ T cells. CFSE dilution profiles of OT1-CD8^+^ T cells after 4 days co-culture with respective DCs without **(A)** or with **(B)** the addition of exogenous ova peptide OVA_257−264_. **(C)** Proportion of OT1-CD8^+^ T cells that have undergone at least one cell division. **(D)** CFSE dilution profiles of donor OT1-CD8^+^ T cells (Ly5.1^+^ and CD8^+^) in the mLN (left). Wild type and Clec9A-DTR recipients were infected with recombinant OT1-PR8 virus 3 h before donor OT1-CD8^+^ T cells transfer (2 × 10^6^), and mLNs were harvested 4 days after infection. Proportion of OT1-CD8^+^ T cells which has either undergone at least one cell division (top right) or no division (bottom right). **(E)** Lungs and mLN were harvested from mice infected with PR8 virus for 10 days. Profiles of intracellular staining for Ki-67 by CD8^+^ T cells in the mLN and lungs. Total number and frequency of Ki-67^+^CD8^+^ T cells in both mLN and lungs. mDC, migratory DC. rDC, resident DC. Data are shown as mean ± SEM. ^**^p < 0.01, ^***^p < 0.001. Data represent two **(C–E)** (*n* = 3–5) independent experiments.

To identify which DC subsets (migratory vs. resident) are involved in cross-presenting IAV antigens, we sorted individual DC subsets (CD103^+^ and CD11b^+^ migratory DCs, and CD8^+^ and CD11b^+^ resident counterparts) from the mLN of mice infected with recombinant IAV (OVA-PR8) and incubated them *in vitro* with CFSE-labeled OT-1 transgenic CD8^+^ T cells for 4–5 days (Supplementary Figures S3A,B). As a control, splenic CD8^+^ and CD11b^+^ cDCs from the same infected mice were sorted and analyzed at the same time (Supplementary Figures S3A,B). Migratory CD103^+^ cDC1s stimulated significantly higher numbers of CD8^+^ T cell divisions (Figure 4A) compared with migratory CD11b^+^ cDC2s (Figure 4A). CD8^+^ T cells co-cultured with either mLN resident CD8^+^ or CD11b^+^ cDCs remained undivided (Figure 4A), comparable with control spleen DC subsets (Figures 4A,C). To exclude cell-intrinsic defects of sorted DC subsets, all DC subsets were shown to display an equal capacity for CD8^+^ T cell activation (Figures 4B,C) when pulsed with exogenous OVA-peptide (OVA_257−264_).

To demonstrate that migratory CD103^+^ cDC1s are important for CD8^+^ T cell activation *in vivo*, CFSE-labeled naïve OT1 CD8^+^ T cells were transferred to both DT-treated wild type and Clec9A-DTR mice, which were previously infected with recombinant OVA-PR8 virus. ~50% of donor OT1 CD8^+^ T cells remained undivided in Clec9A-DTR mLNs whereas only 20% of OT1 CD8^+^ T cells were undivided in wild type mLN (Figure 4D). Thus, optimal expansion of naïve CD8^+^ T cells *in vivo* requires migratory CD103^+^ cDC1s during IAV infection. To visualize proliferating CD8^+^ T cells, mLNs and lungs from PR8-infected wild type and Clec9A-DTR mice were collected 10 days post infection and stained for the transcription factor, Ki-67, in CD8^+^ T cells (Figure 4E). The frequency and total numbers of Ki-67^+^ CD8^+^ T cells in the mLNs were significantly lower in Clec9A-DTR mice compared with wild type mice (Figure 4E). However, in the lungs, no difference between groups was observed, indicating that CD103^+^ cDC1s do not support CD8^+^ T cell proliferation within the lung itself. Overall, our data demonstrate that migratory CD103^+^ cDC1s are required for the optimal expansion of antigen-specific CD8^+^ T cells in the mLN (Figures 4A–E).

### CD103^+^ cDC1s Support the LN Egression of IAV-Specific CD8^+^ T Cells

**Figure 5:**
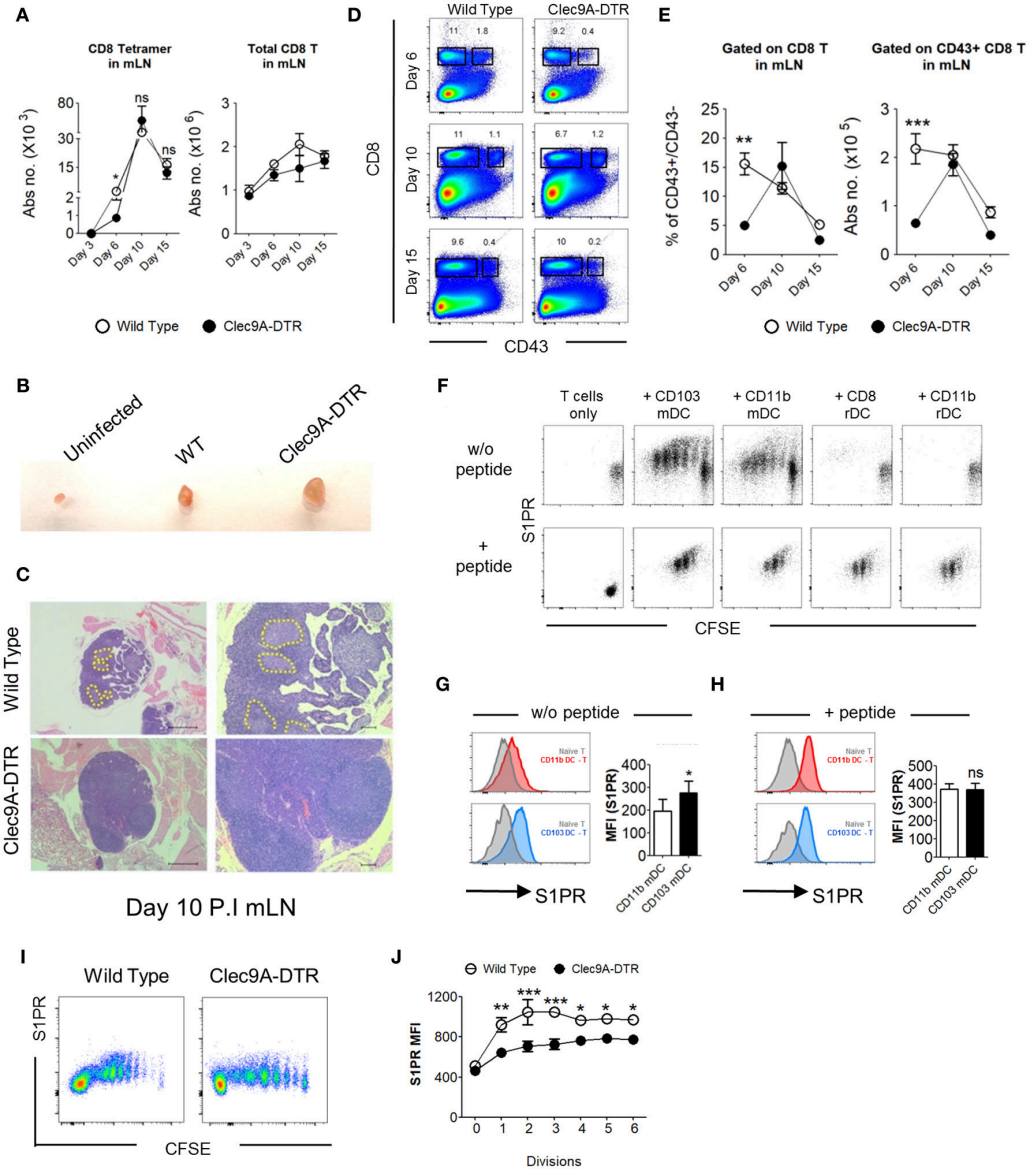
CD103^+^ cDC1s support the LN egression of IAV-specific CD8^+^ T cells. **(A)** Total number of NP_366−374_-specific CD8^+^ T cells (left) and bulk CD8^+^ T cells (right) in the mLN of wild type and Clec9A-DTR mice on day 3, 6, 10, and 15 of infection. **(B)** Representative picture of posterior mLN harvested from uninfected mice and infected Clec9A-DTR and wild type mice after 10 days of infection. **(C)** Representative H&E histology images of posterior mLN harvested from day 10-infected Clec9A-DTR and wild type mice. **(D)** Representative flow cytometry profiles of CD8 and CD43 stained cells obtained from mLN harvested on day 6, 10, and 15 of infection. **(E)** Frequency of CD43^+^/CD43^−^ CD8^+^ T cells in the mLN (left). Total number of CD43^+^ CD8^+^ T cells in the mLN (right) on day 6, 10, and 15 of infection. **(F)** Flow cytometry profiles of OT1^+^CD8^+^ T cells co-cultured with sorted DCs for 3 days with or without exogenous SIINFEKL peptide. Cells are gated on Ly5.1^+^, CD8^+^, and CD3^+^. **(G)** MFI of S1PR by OT1^+^CD8^+^ T cells that were co-cultured without DC (gray filled), with migratory CD103^+^ cDC1s (blue), or with migratory CD11b^+^ cDC2s (red) in the absence of exogenous SIINFEKL peptide. **(H)** MFI of S1PR by OT1^+^CD8^+^ T cells that were co-cultured without DC (gray filled), with migratory CD103^+^ cDC1s (blue), or with migratory CD11b^+^ cDC2s (red) in the presence of exogenous SIINFEKL peptide. **(I)** CFSE dilution profiles of donor OT1-CD8^+^ T cells in the mLN. Wild type and Clec9A-DTR recipients were infected with recombinant OVA-PR8 virus 3 h before donor OT1-CD8^+^ T cells transfer (2 × 10^6^), and mLNs were harvested 4 days after infection. **(J)** MFI of S1PR expression by donor OT1-CD8^+^ T cells in each cell division. Data are shown as mean ± SEM. ^*^p < 0.05. ^**^p < 0.01. ^***^p < 0.001. Data represent two **(A,E,G–J)** (*n* = 3–5) independent experiments.

Because virus-specific CD8^+^ T cell activation and expansion is critically dependent on migratory CD103^+^ cDC1s, it might be expected that the number of NP_366−374_-specific CD8^+^ T cells would be reduced in the mLN of Clec9A-DTR mice. However, the numbers of NP_366−374_-specific CD8^+^ T cells, as well as total CD8^+^ T cells, in the mLN of Clec9A-DTR mice were comparable to those in wild type mice (Figure 5A). In addition, the mLN from the IAV-infected Clec9A-DTR mice were clearly enlarged, with less distinguishable T cell zones and B cell follicles compared with wild type mice (Figures 5B,C). CD43 was upregulated in NP_366−374_-specific CD8^+^ T cells in the mLN (Supplementary Figure S4A). Therefore, we used CD43 as a marker to distinguish activated from non-activated CD8^+^ T cells. The ratios of CD43^+^ to CD43^−^CD8^+^ T cells, as well as total CD43^+^ CD8^+^ T cells, were significantly lower in Clec9A-DTR mLN on day 6 post infection, but not on day 10 and 15 (Figures 5D,E). Because reduced proliferation was detected at day 10 post infection (Figure 4E), we speculated that the augmented numbers of activated LN CD8^+^ T cells in Clec9A-DTR mice from this time point may be related to a lack of egression from the LN. To test this, we focused on possible chemokine receptors involved in T cell trafficking. Sphingosine-1-phosphate receptor (S1PR) is important for the egression of lymphocytes from the mLN; therefore, we hypothesized that the ablation of migratory CD103^+^ cDC1s may affect S1PR expression on activated CD8^+^ T cells, resulting in their accumulation in the mLN. We infected wild type mice with recombinant OVA-PR8 and harvested the mLN after 5 days. Individual DC subsets were sorted from the mLN and co-cultured with OT1-CD8^+^ T cells for 3 days before analysis of S1PR expression levels on CD8^+^ T cells (Figure 5F). CD8^+^ T cells co-cultured with migratory CD103^+^ cDC1s expressed significantly higher levels of S1PR compared to those co-cultured with CD11b^+^ DC subsets, which minimally upregulated this receptor (Figures 5F,G) suggesting that effector CD8^+^ T cells, primed and differentiated in the mLN, require migratory CD103^+^ cDC1s for efficient LN egression. In the presence of exogenous OVA_257−264_ peptide, the expression level of S1PR on the OT1-CD8^+^ T cells appeared to be similar regardless of the DC subset used for co-culturing (Figures 5F,H), suggesting that S1PR expression on CD8^+^ T cells is related to the efficiency of antigen presentation rather than a cell-intrinsic difference. To examine whether S1PR regulation by CD103^+^ cDC1s can be similarly observed *in vivo*, we transferred OT1-CD8^+^ T cells to both recombinant OVA-PR8 virus infected wild type and Clec9A-DTR mice and analyzed the S1PR expression by donor OT1-CD8^+^ T cells 4 days after. We observed that in the absence of CD103^+^ cDC1s, donor OT1-CD8^+^ T cells failed to strongly upregulate the S1PR expression (Figures 5I,J). This confirms the *in vitro* data that CD103^+^ cDC1s are involved in the induction of S1PR expression by CD8^+^ T cells.

To understand whether the reduced pulmonary CTL population in Clec9A-DTR mice can be contributed by defective CD8^+^ T cell entry to lung, we profiled influenza-specific CD8^+^ T cells in the peripheral blood (Supplementary Figure S4B). In Clec9A-DTR mice the circulating NP_366−374_- specific CD8^+^ T cells were significantly lower than in wild type mice indicating that these cells do not accumulate in the blood. Therefore, an impairment of CD8^+^ T cell lung entry may not be affected by CD103^+^ cDC1 ablation (Supplementary Figure S4B). Since two chemokine receptors VLA-1 and CXCR3 have been proposed to regulate lung entry of CD8^+^ T cells (5, 17–19), we examined the surface expression of VLA-1(CD49a, α-chain of VLA-1) and CXCR3 on the CD8^+^ T cells and have found that regardless of the presence or absence of CD103^+^ cDC1s, the expression of VLA-1(CD49a) and CXCR3 was not significantly affected. Thus, CD103^+^ cDC1s may not be responsible for chemokine receptors imprinting for CD8^+^ T cells lung entry (Supplementary Figure S4C).

### CD103^+^ cDC1s Alter Effector CD8^+^ T Cell Survival in IAV-Infected Lungs

**Figure 6:**
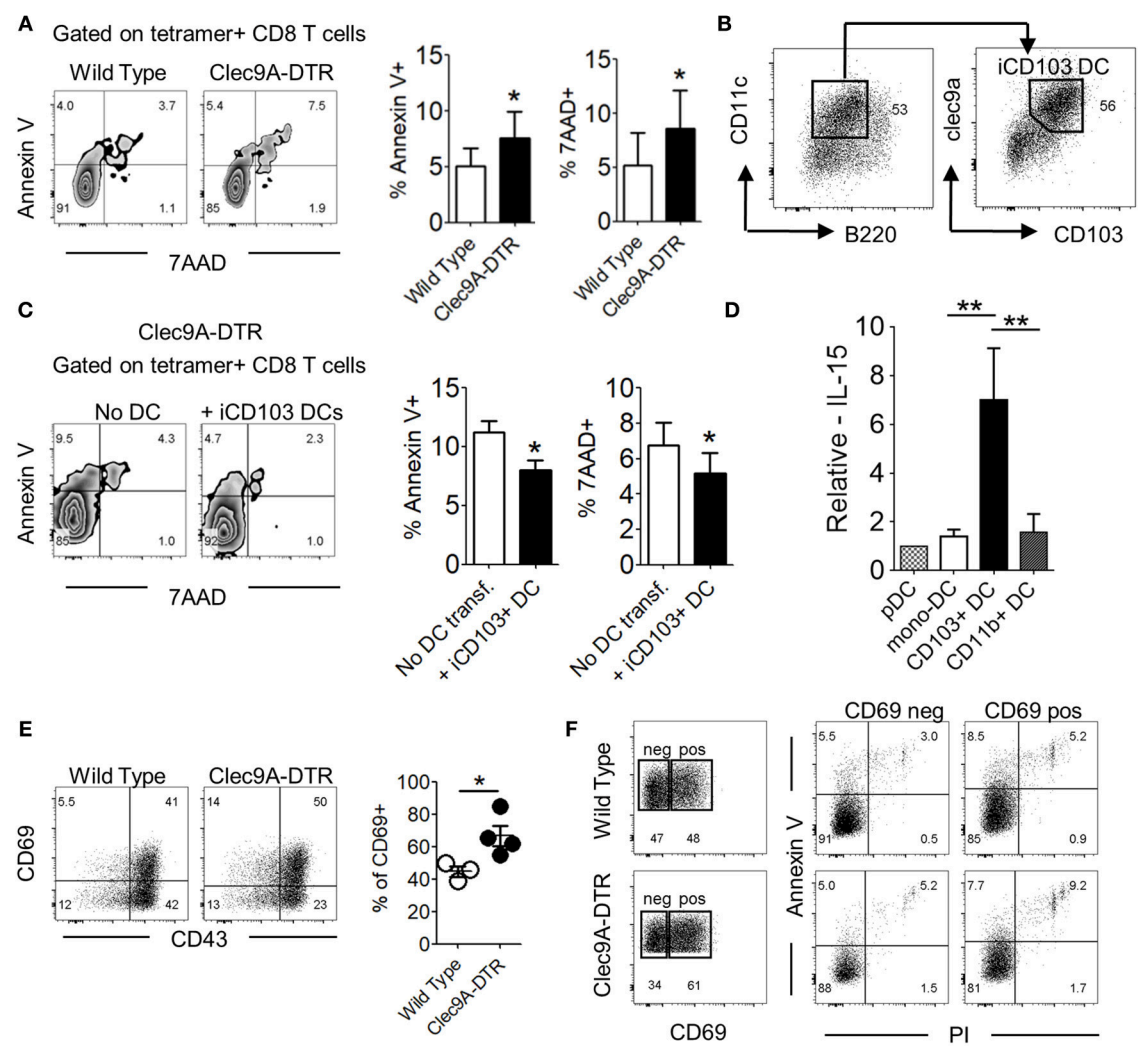
CD103^+^ cDC1s alter effector CD8^+^ T cell survival in IAV-infected lungs. **(A)** Annexin V and 7AAD staining profile of NP_366−374_-specific CD8^+^ T cells in the lung of wild type and Clec9A-DTR mice on day 10 of infection (left). Frequency of Annexin V^+^/7AAD^+^ NP_366−374_-specific CD8^+^ T cells (right). **(B)** Characterization of BM-differentiated CD103^+^ cDC1s (iCD103^+^ cDC1s) after 16 days in culture supplemented with GM-CSF and Flt3L. **(C)** Annexin V and 7AAD staining profile of NP_366−374_-specific CD8^+^ T cells in the lung of Clec9A-DTR recipient mice with or without adoptive transfer of iCD103^+^ cDC1s on day 11 infection (left). Frequency of Annexin V^+^/7AAD^+^ NP_366−374_-specific CD8^+^ T cells (right). **(D)** Relative mRNA expression of IL-15 by plasmacytoid DCs, monocyte-derived DCs, migratory CD103^+^ cDC1s, and migratory CD11b^+^ cDC2s from infected wild type mice on day 10 infection. **(E)** Representative CD69 and CD43 staining profile of NP_366−374_-specific CD8^+^ T cells in the lung of wild type and Clec9A-DTR mice on day 10 of infection (left) and calculated frequencies of CD69^+^ NP_366−374_-specific CD8^+^ T cells (right). **(F)** Representative CD69^+^ and CD69^−^ gates on NP_366−374_-specific CD8^+^ T cells in the lung of wild type and Clec9A-DTR mice on day 10 infection (left) and representative surface expression profiles of Annexin V^+^ and PI^+^ by CD69^+^ and CD69^−^ populations. Data are shown as mean ± SEM. ^*^p < 0.05. ^**^p < 0.01. Data represent two **(A,C–F)** (*n* = 3–5) independent experiments.

Effector CD8^+^ T cells in influenza-infected lungs are replenished by constant cell recruitment from the mLN and undergo a second wave of expansion upon arrival in the lungs (4). Cytotoxic CD8^+^ T cells are actively maintained by local signals e.g., IL-15 (20). We observed that, beyond a reduced total number of virus-specific CD8^+^ T cells in Clec9A-DTR mice, higher proportions of these cells were not viable, indicating that CD103^+^ cDC1s may be required for the maintenance and/or survival of this effector population (Figure 6A and Supplementary Figure S5C). To investigate the involvement of CD103^+^ cDC1s in CD8^+^ T cell viability, we generated CD103^+^ cDC1s from *in vitro* BM cultures (Figure 6B). Purified cDC1s were transferred into Clec9A-DTR mice on day 6 post infection and lungs were harvested 4 days later (Supplementary Figures S5A,B). The viability of virus-specific CD8^+^ T cells harvested from Clec9A-DTR mice significantly improved following transfer of CD103^+^ cDC1s, indicating a direct function of CD103^+^ cDC1s in promoting survival of virus-specific effector CD8^+^ T cells (Figure 6C). To identify possible factors promoting CD8^+^ T cell survival, distinct pulmonary myeloid cell subpopulations (pDCs, monocyte-derived DCs, CD103^+^ cDC1s, and CD11b^+^ cDC2s) were isolated from IAV-infected lungs, purified by cell sorting, and analyzed for IL-15 expression, a cytokine involved in the maintenance of naïve and memory T cell populations (20–22). IL-15 transcript levels were significantly higher in CD103^+^ cDC1s compared with other subsets, indicating that this particular DC subset is likely to be involved in supporting virus-specific CD8^+^ T cell survival in the lung via IL-15 (Figure 6D). Moreover, in the absence of CD103^+^ DCs the pulmonary IL-15 transcript levels were lower when compared to normal lungs, confirming their contribution in the production of this particular cytokine (Supplementary Figure S5D).

Activation-induced cell death (AICD) leads to the contraction of effector T cell populations upon pathogen clearance (23). We noted that a higher proportion of NP_366−374_-specific CD8^+^ T cells in the Clec9A-DTR group expressed the activation marker, CD69, compared with those found in the wild type group on day 10 of infection (Figure 6E). The higher frequency of CD69^+^ effector CD8^+^ T cells in Clec9A-DTR mice was due to higher pulmonary virus load. However, viability of the CD69^+^CD8^+^ T cell population was lower compared with its CD69^−^CD8^+^ T cell counterpart, suggesting that AICD contributes to the lower cell viability of CTLs in Clec9A-DTR mice (Figure 6F).

### CD103^+^ cDC1s Are Essential for Cross-Reactive Protection Against Serotypically Distinct Virus Subtypes

**Figure 7:**
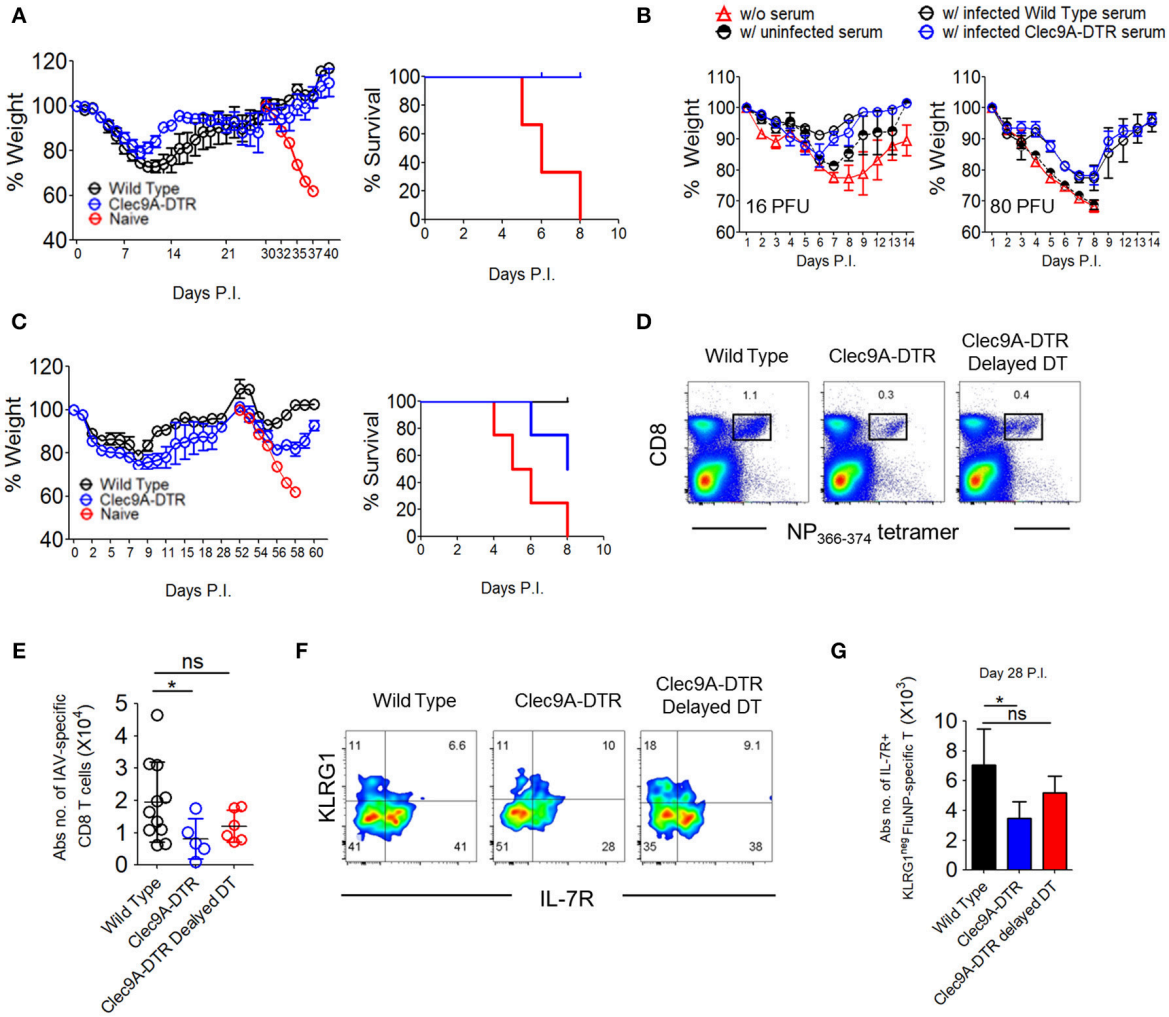
CD103^+^ cDC1s are essential for cross-reactive protection against serotypically distinct virus subtypes. **(A)** Wild type and Clec9A-DTR mice were infected with 3 PFU x-31 virus and subsequently infected with 600 PFU x-31 virus after 30 days. Naïve mice were infected with 600 PFU x-31 virus on day 30 only. Weight loss curve (left) and survival curve (right) for mice after 600 PFU x-31 virus infection. **(B)** Sera were harvested from naïve, infected wild type, and Clec9A-DTR mice. Wild type recipient mice were administered with 50 μl of collected serum 2 days before infection with 16 or 80 PFU PR8 virus. Wild type mice without serum transfer were used as controls. Weight loss curve of recipients which received 16 PFU (left) and 80 PFU (right) PR8 virus. **(C)** Wild type and Clec9A-DTR mice were infected with 3 PFU x-31 virus and subsequently infected with 600 PFU PR8 virus after 28 days. Naïve mice were infected with 600 PFU PR8 virus on day 28 only. Weight loss curve (left) and survival curve for mice after 600 PFU PR8 virus infection (right). **(D,E)** Representative flow cytometry plots and absolute numbers of NP_366−374_-specific CD8^+^ T cells in the lungs of wild type, Clec9A-DTR and Clec9A-DTR delayed DT injected mice on day 35 of infection. Clec9A-DTR mice were injected with DT from day−5 to day 14 of infection. Clec9A-DTR delayed DT mice were injected with DT from day 21 to day 35 of infection. **(F)** Representative flow cytometry staining of KLRG1 and IL7R expression by NP_366−374_-specific CD8^+^ T cells (day 35 P.I). **(G)** Absolute number of IL7R^+^ NP_366−374_-specific CD8^+^ T cells in the lungs (day 35 P.I). Data are shown as mean ± SEM. ^*^p < 0.05. Data represent two [**(A,B)** (*n* = 3–5) and **(C,E,G)** (*n* = 3–4)] independent experiments.

A host infected with a particular influenza virus subtype (defined by virus surface protein—Hemagglutinin [HA] and Neuraminidase [NA]), is unlikely to be re-infected by a virus of same HA/NA subtype due to influenza-specific antibodies. We therefore examined whether such antibody-mediated memory protection, termed homosubtypic immunity, requires migratory CD103^+^ cDC1s (Supplementary Figure S6A). All X-31 pre-immunized control and Clec9A-DTR mice recovered from a secondary challenge using a lethal X-31 virus dose (Figure 7A), suggesting that ablation of CD103^+^ cDC1s does not significantly impact antibody responses. This infection set-up will also trigger T cell response if the virus-specific antibodies in the pre-immunized mice do not fully neutralize all virus particles. Because both pre-immunized wild type and Clec9A-DTR mice did not lose weight after secondary lethal challenge, it appears that virus-neutralizing antibodies remove most virus particles suggesting that the hosts do not require a highly robust T cell response to eliminate the “escaped” virus particles. Next, we harvested sera from PR8 pre-immunized control and Clec9A-DTR mice and transferred to naïve recipients 2 days before infecting them with either 16 or 60 PFU dose of PR8. Sera from either pre-immunized wild type or Clec9A-DTR mice conferred similar protection to naïve recipients, providing further evidence that homosubtypic immunity does not require migratory CD103^+^ cDC1s (Figure 7B and Supplementary Figure S6B).

To investigate the involvement of cDC1s in heterosubtypic immunity, we first infected both wild type and Clec9A-DTR mice with a sublethal dose of X-31 virus and housed mice for 4–5 weeks before secondary challenge with the serotypically distinct PR8 virus (Supplementary Figure S6C). Re-challenged wild type mice initially lost weight during the 1st week of secondary PR8 infection and then recovered steadily to full body weight. In contrast, 50% of Clec9A-DTR mice succumbed to the secondary challenge after displaying a similar initial weight loss over the 1st week. This observation suggests that CD103^+^ cDC1s are required to generate efficient CD8^+^ T cell-mediated memory protection (Figure 7C).

To understand whether reduced memory T cells are responsible for increased susceptibility of Clec9A-DTR mice to secondary challenge, we enumerated the number of virus-specific CD8^+^ T cells 5 weeks after the infection during which memory effector cell populations are typically present (24). One group of wild type mice and two groups of Clec9A-DTR mice were examined, one Clec9A-DTR mouse group was treated DT for 1st 3 weeks (Clec9A-DTR group) while second group was treated DT on the 4th and 5th week (Clec9A-DTR delayed DT group). Compared to wild type mice, we observed significantly diminished numbers of virus-specific CD8^+^ T cells in Clec9A-DTR mice 5 weeks post infection (Figures 7D,E). Using IL-7R and KLRG1 surface expression profiling as a marker of antigen experience, IL-7R^+^KLRG1^−^ memory effector CD8^+^ T cells were indeed reduced in Clec9A-DTR mice (Figures 7F,G). These results demonstrate that the diminished protection from lethal secondary challenge observed in Clec9A-DTR mice may be due to the reduced number of memory CD8^+^ T cells (Figure 7C). Clec9A-DTR delayed DT group allows us to assess whether CD103^+^ cDC1s are required to maintain the memory effector CD8^+^ T cell pool, we omitted DT injections during the first 3 weeks of infection to allow effective primary effector antiviral CD8^+^ T cell response. DT was injected only during the 4th and 5th weeks post infection, and mice were killed at the end of the 5th week. A slight, but non-significant, decrease in the number of memory effector CD8^+^ T cells was measured in Clec9A-DTR delayed DT group compared with wild type mice, indicating that CD103^+^ cDC1s do not impact significantly on the development and maintenance of memory effector CD8^+^ T cells (Figures 7D–G) once an optimal primary effector CD8^+^ T cell response is generated.

## Discussion

It is now well-established that cDC1s are essential for effective influenza immunity (1, 6, 12). Consistent with this, the Clec9A-DTR mouse, an *in vivo* depletion model of CD103^+^ cDC1s, displays increased IAV susceptibility, confirming the importance of this migratory pulmonary DC subset during IAV infection. The decreased resistance observed in the Clec9A-DTR mouse line correlates with a significantly reduced CD8^+^ T cell response and increased viral burden, leading to extensive lung inflammation and tissue damage (1, 6, 12). Because the accumulation of CTLs in the infected lung governs viral clearance, a better understanding of the distinct checkpoints regulating the priming, expansion, migration, and survival of these CTLs is crucial for the development of strategies aimed at improving host resistance.

CD103^+^ cDC1s have been widely recognized for their effective cross-presentation capacity, not only during the immune response to infection (including to IAV) (1) but also in anti-tumor immunity (25, 26). Pulmonary CD103^+^ cDC1s are resistant to IAV infection via a type I interferon-mediated anti-viral state and are not only able to carry viral antigens to the draining LNs but also to cross-present them efficiently to naïve CD8^+^ T cells in the mLN (13). Here, using the Clec9A-DTR mouse model, we reconfirm that CD103^+^ cDC1s are the predominant DC subset required for the expansion of antigen-cognate CD8^+^ T cells in the mLN during IAV infection. Non-migratory resident CD8^+^ cDC1s have also been suggested to function in viral antigen cross presentation in the mLN (27); however, we find that CD103^+^ cDC1s account for the vast majority of viral antigen cross presentation, both *in vivo* and *in vitro*, whereas the contribution of migratory and resident CD11b^+^ cDC2s and resident CD8^+^ cDC1s is minimal. Nonetheless it has been shown previously that the non-migratory resident CD8^+^ cDC1s are capable of cross-present virus antigen to naïve CD8^+^ T cells, this is only possible when they acquire virus antigen from the migratory CD103^+^ cDC1s (27). Therefore, CD103^+^ cDC1s aside from efficiently cross-present acquired virus-antigen to naïve CD8^+^ T cells, they also act as a source of virus antigen to the resident DCs.

During influenza infection the expansion of CD8^+^ T cells occurs not only in mLN but also in the lung (4). It has been reported that expansion of pulmonary CD8^+^ T cells contributes significantly to the accumulation of these cells even though there is ongoing cell recruitment from the mLN (4). The CTL expansion in lungs requires both antigen and costimulation to sustain the active cell division which is controlled by DCs (28). In contrast to the predominant role of CD103^+^ cDC1 in cross-priming naïve CD8^+^ T cells in the mLN, proliferation of effector CD8^+^ T cells in the lung can be driven by a number of other DC subpopulations (29). The dispensability of pulmonary CD103^+^ cDC1 for the induction of local CTLs active division corroborates with our observation that the number of proliferating CTLs in the lung are unaffected in the absence of CD103^+^ cDC1s.

Furthermore, our results demonstrate that the function of CD103^+^ cDC1s is not restricted to antigen cross presentation in the LN compartment, but extends to other key factors required for effective viral clearance by CTLs. As such, CD103^+^ cDC1s promote CD8^+^ T cell LN egression during early IAV infection, as well as pulmonary CD8^+^ T cell survival. Importantly, particularly in the context of vaccination strategies, this particular DC subset also contributes to the development of memory CTLs, which is important for the acquisition of broad spectrum cross-protection against multiple IAV serotypes.

During an effective anti-viral response, antigen-specific CD8^+^ T cells expand in the lung-draining mLN and traffic back to the site of active infection, the lungs in this case, where they effectively clear the viral infection. This migration is regulated by chemokine receptors, such as S1PR, that control the accumulation of effector T cells in the peripheral organs (30). Our results show that, during the progression of IAV infection, migratory CD103^+^ cDC1s modulate the expression of S1PR on CD8^+^ T cells in the mLN, promoting the egression of activated CD8^+^ T cells. Coupled with the superior capacity of CD103^+^ cDC1s to cross-prime naïve CD8^+^ T cells on a per-cell basis, they are therefore uniquely important for the expansion and trafficking of cognate CD8^+^ T cells during IAV infection. Accumulation of LN CD8^+^ T cells in the absence of CD103^+^ cDC1s has been previously reported. Kim et al. demonstrated that CD103^+^ cDC1s, but not CD11b^+^ cDC2s, promoted CD8^+^ T cell lung homing due to differential expression of CD24 (31). Another study similarly reported that absence of CD103^+^ cDC1s downregulated effector CD8^+^ T cells in the lung but not in the LN during late infection stage (32). Here, we demonstrate that CD103^+^ cDC1s induce S1PR expression on antigen-specific CD8^+^ T cells, as a consequence of the numbers of antigens displayed on the cell surface, rather than a cell-intrinsic difference. In contrast, their migratory CD11b^+^ cDC2 counterparts are more prominently involved in supporting the proliferation of CD8^+^ T cells in a CD70-dependent manner rather than cross-presenting antigen to naïve CD8^+^ T cells (2). The distinct hierarchy of migratory CD103^+^ cDC1s and CD11b^+^ cDC2s in antigen cross presentation is thought to be a result of their differential responses to type I IFN signaling (13, 33). In addition, the selective expression of Clec9A (also known as DNGR-1) on CD103^+^ cDC1s allows dead-cell antigen binding/uptake for recycling endosomal route favoring robust antigen cross presentation (34, 35).

In addition to CTL homing to the site of infection, CTL survival within the infected tissue via cytokine signaling is essential to control IAV infection (21). It was previously shown that IL-15 signaling is key to promote the survival of CD8^+^ T cells in the lung, a role fulfilled by pulmonary DCs (20). The significantly higher numbers of apoptotic CD8^+^ T cells in Clec9A-DTR mice might therefore be due to the absence of IL-15-producing CD103^+^ cDC1s. The markedly reduced pulmonary virus-specific CD8^+^ T cell fraction not only leads to persistence of the virus in the lungs, but also to the impairment of an effective memory CD8^+^ T cell population at the site of infection. In an elegant study by Shen et al., it was shown that the developmental potential of memory T cells is both time-sensitive and time-dependent on the number of virus antigen-bearing DCs (36), hence disease progression has a substantial and significant impact on the memory fate decision of these primary effector cells. Our observations similarly suggest that the lack of memory CD8^+^ T cells in Clec9A-DTR mice is a result of altered disease progression caused by delayed viral clearance. Thus, the absence of CD103^+^ cDC1s impacts not only on the primary CD8^+^ T cell effector population, but also its memory T cell development. Furthermore, the higher expression of CD69 by Clec9A-DTR CD8^+^ T cells, most likely due to higher pulmonary virus titers in the absence of cDC1s (18), indicates that these effector cells are repeatedly activated and become more exhausted and more prone to apoptosis during the contraction phase (18, 37), hampering their ability to become memory precursors.

Recent reports on LN-resident DCs (CD8^+^ cDC1) strongly advocate their role as a platform for CD4^+^ T cells in augmenting memory CD8^+^ T cell formation, recall and fitness in virus infection models (38–40). Considering that in Clec9A-DTR mice also resident CD8^+^ cDC1s are affected, the lack of memory CD8^+^ T cell response in these mice during influenza re-infection can be a combination of insufficient CD4^+^ T cell help and the lack of immunogenic CTL priming. It is widely reported that CD4^+^ T cells, though dispensable for primary CTL response, are central to achieve robust memory CD8^+^ T cell response in influenza infection (41–44). Therefore, it cannot formally be excluded that the reduced protection observed in the Clec9A-DTR mouse during heterologous re-infection is a consequence of diminished CD4^+^ T cell mediated-support for memory CTL recall (42).

Recent progress in identifying human CD141c^+^ DC as functional homolog of murine CD103^+^ cDC1 has since allowed inference to be drawn between mouse and human studies (45). Effort in humanized mouse studies has subsequently demonstrated functional correlations between murine and human DCs in influenza infection (46). In light of these reports, insights obtained from mouse CD103^+^ cDC1 functional study would thus be important toward the understanding of human CD141c^+^ DC.

In summary, we identify previously unappreciated contributions of CD103^+^ cDC1s in the regulation of a strong and effective influenza-specific CTL response. Beyond their established role as antigen cross-presenting cells, CD103^+^ cDC1s facilitate effector T cell egression from the LN and promote T cell survival in the lungs, both important events for effector T cell accruement at the site of infection. Our observation that CD8^+^ T cell-dependent cross-reactive immunoprotective memory requires an intact CD103^+^ cDC1 population strongly suggests that vaccination strategies aimed at boosting anti-viral T immunity should consider the modulation of cDC1s to achieve optimal outcomes.

## Author Contributions

SLN and CR: conceptualization; SLN, YJT, and YAS: methodology and investigation; SLN: formal analysis; SLN: writing—original draft; CR: writing—review and editing and funding acquisition; KK and CR: supervision.

### Conflict of Interest Statement

The authors declare that the research was conducted in the absence of any commercial or financial relationships that could be construed as a potential conflict of interest.
