# Supplementary material for: Type 1 Conventional CD103+ Dendritic Cells Control Effector CD8+ T Cell Migration, Survival, and Memory Responses During Influenza Infection
Source: Front Immunol. 2018 Dec 21;9:3043. doi: 10.3389/fimmu.2018.03043 (PMC6308161; doi:10.3389/fimmu.2018.03043)
Supplement: Supplementary file 1 [file Data_Sheet_1.PDF]

mLN

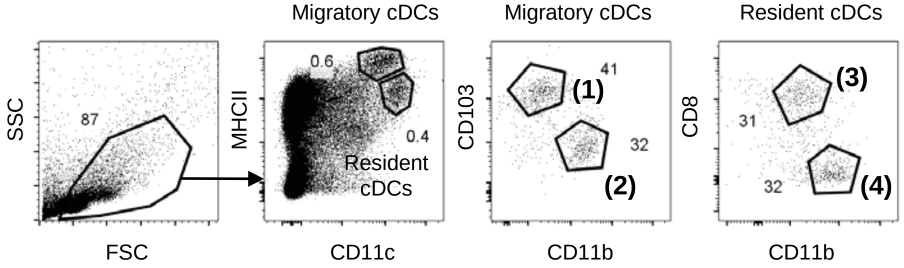

(1) migratory CD103$^+$ cDC1
(2) migratory CD11b$^+$ cDC2
(3) resident CD8$^+$ cDC1
(4) resident CD11b$^+$ cDC2

Supplementary Fig. 1: Gating strategy for migratory CD103$^+$ cDC1, migratory CD11b$^+$ cDC2, resident CD8$^+$ cDC1, and resident CD11b$^+$ cDC2 in the mLN of an uninfected mouse.

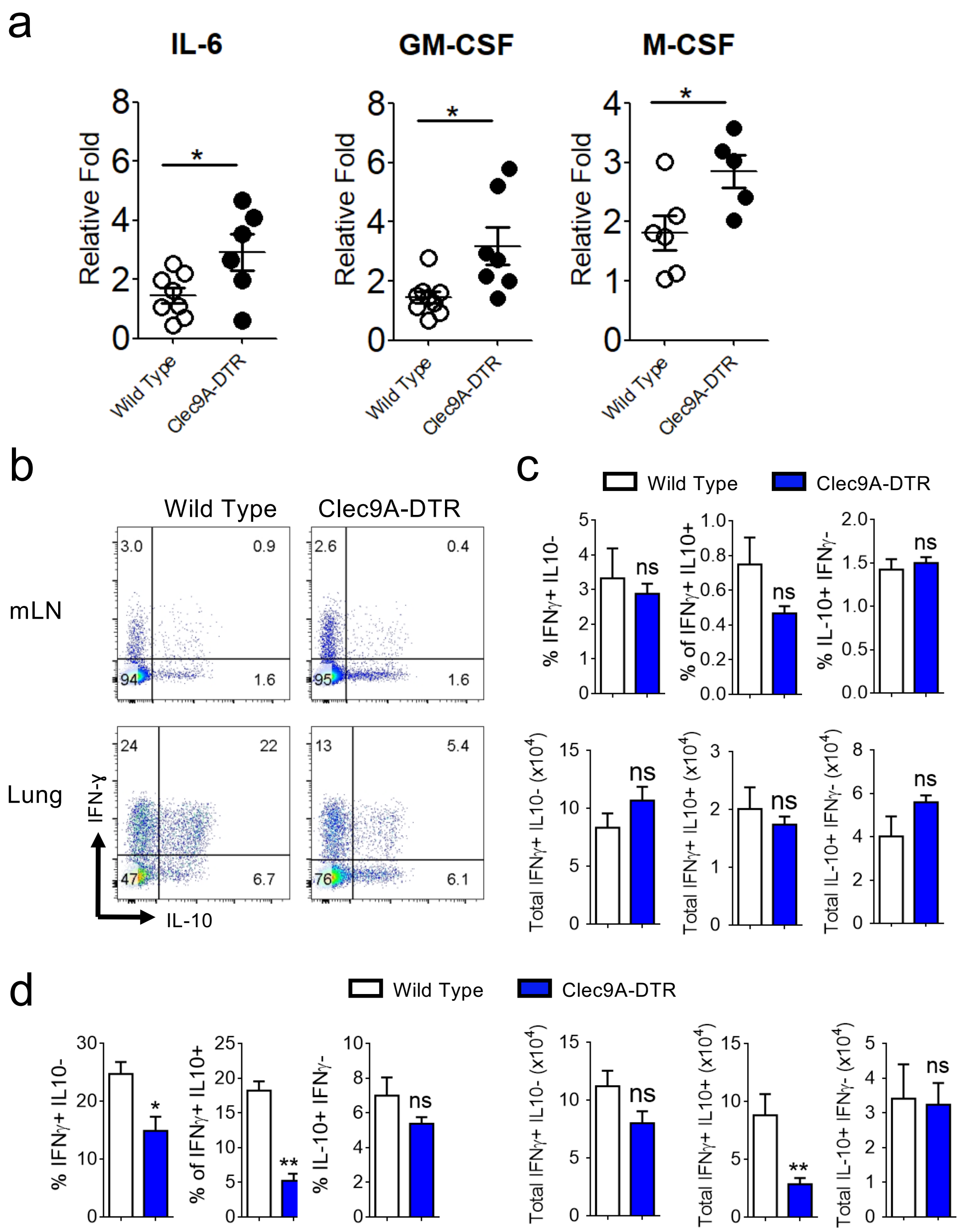

Supplementary Fig. 2: (a) Quantitative real-time PCR for IL-6, GM-CSF, and M-CSF in lung tissue from wild type and Clec9a-DTR mice after 10 days of infection. (b-d) Lung and mLN were harvested from uninfected, infected wild type, and Clec9A-DTR mice on day 10 post infection and stimulated with PMA/Iono for 3 h followed by Brefeldin A incubation for an additional 3 h. Intracellular IFN-γ and IL-10 staining profiles of pulmonary and mLN $CD4^+$ T cells (b), frequency and total IFN-γ-producing, IL-10-producing, and IFN-γ double-producing $CD4^+$ T cells in the mLN (c), frequency and total IFN-γ-producing, IL-10/-producing, and IFN-γ/IL-10 double-producing $CD4^+$ T cells in the lung (d). Data are shown as mean ± SEM. *, $p < 0.05$. **, $p < 0.01$. Data represent two (n = 4) independent experiments.

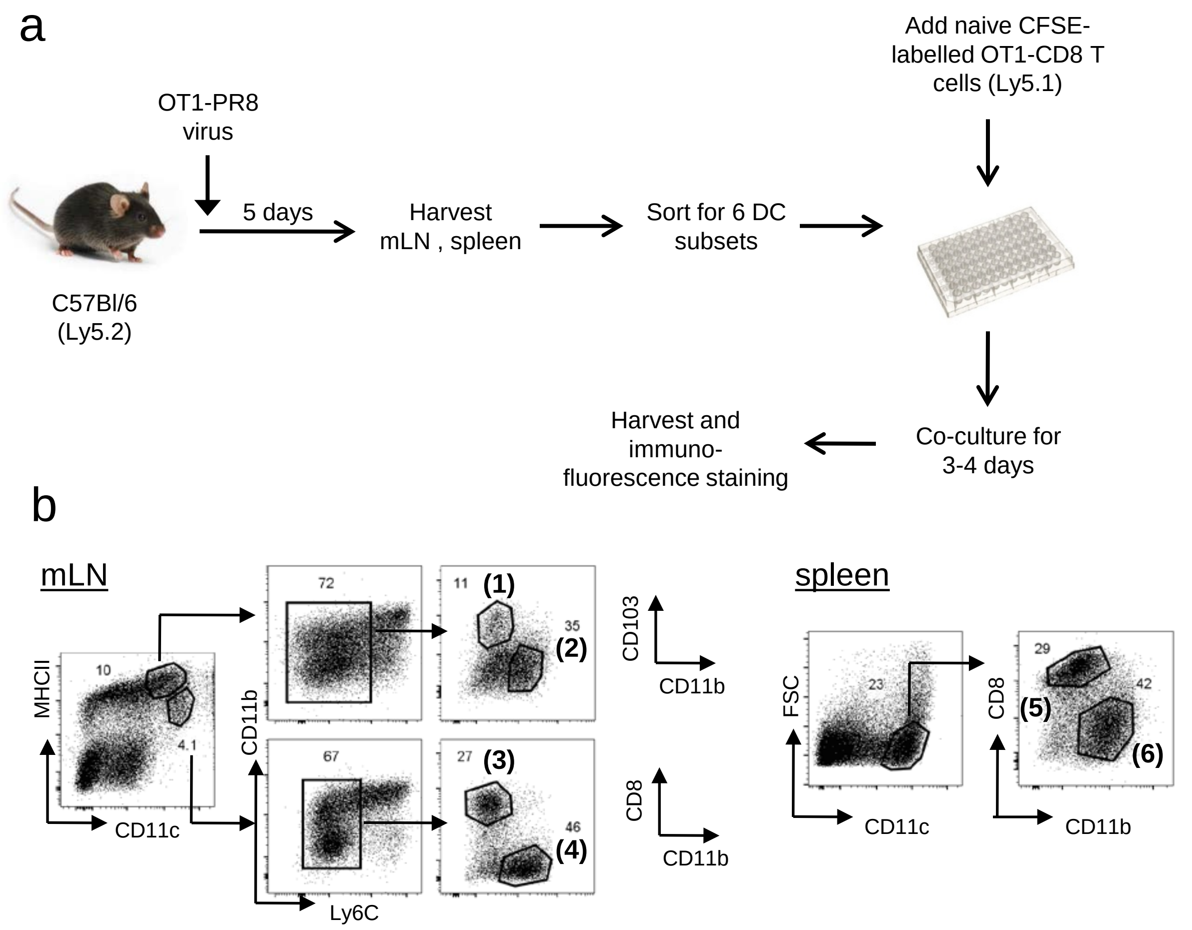

mLN and Spleen DCs which are sorted:

(1) migratory $CD103^+$ cDC1
(2) migratory $CD11b^+$ cDC2
(3) resident $CD8^+$ cDC1
(4) resident $CD11b^+$ cDC2
(5) $CD8^+$ cDC1
(6) $CD11b^+$ cDC2

Supplementary Fig. 3: (a) Wild type C57BL/6 mice were infected with recombinant OT1-PR8 virus for 5 days before harvesting the spleens and mLNs. $CD8^+$ cDC1 and $CD11b^+$ cDC2 from the spleen, together with migratory $CD103^+$ cDC1, migratory $CD11b^+$ cDC2, resident $CD8^+$ cDC1, and resident $CD11b^+$ cDC2 from the mLN, were sorted and subsequently co-cultured with CFSE-labeled naïve $CD8^+$ T cells from OT1 transgenic mice. (b) Gating strategy for the sorting of DCs in mLN and spleen.

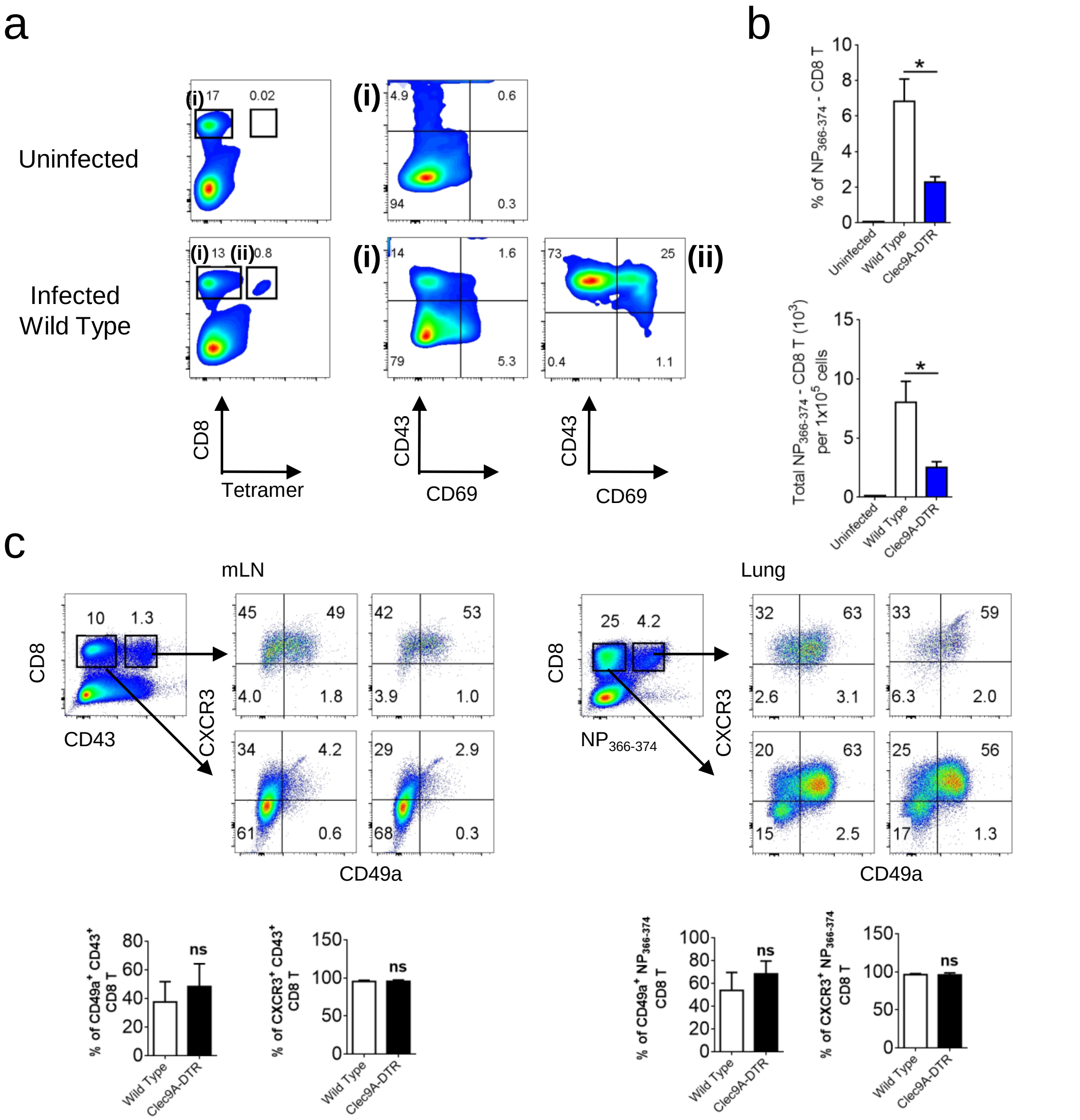

Supplementary Fig. 4: (a) mLNs from uninfected and infected wild type mice (day 10 post infection) were stained for $NP_{366\text{-}374}$ -tetramer, CD8, CD43, and CD69. $CD8^{+}$ T cells form uninfected mice were negative for $NP_{366\text{-}374}$ –tetramer staining. Infected wild type mice $CD8^{+}$ T cells, which stained positive for $NP_{366\text{-}374}$ –tetramers, co-express CD43 (ii). A subset of $NP_{366\text{-}374}$ – $CD8^{+}$ T cells co-express CD69 (ii). (b) Frequency and number of $NP_{366\text{-}374}$ – specific CD8 T cells in the blood harvested from uninfected, infected wild type and Clec9A-DTR mice on day 10 of infection. (c) Profile of CXCR3 and CD49a surface staining for activated $CD8^{+}$ T cells in the mLN and $NP_{366\text{-}374}$-specific $CD8^{+}$ T cells in the lung (top), frequency of ($CD49a^{+}$/$CXCR3^{+}$) $CD43^{+}CD8^{+}$ T cells in the mLN and frequency of ($CD49a^{+}$/$CXCR3^{+}$) $NP_{366\text{-}374}$-$CD8^{+}$ T cells in the lung (bottom) on day 10 of infection. Data are shown as mean ± SEM. *, $p < 0.05$. Data represent two (n = 4) independent experiments.

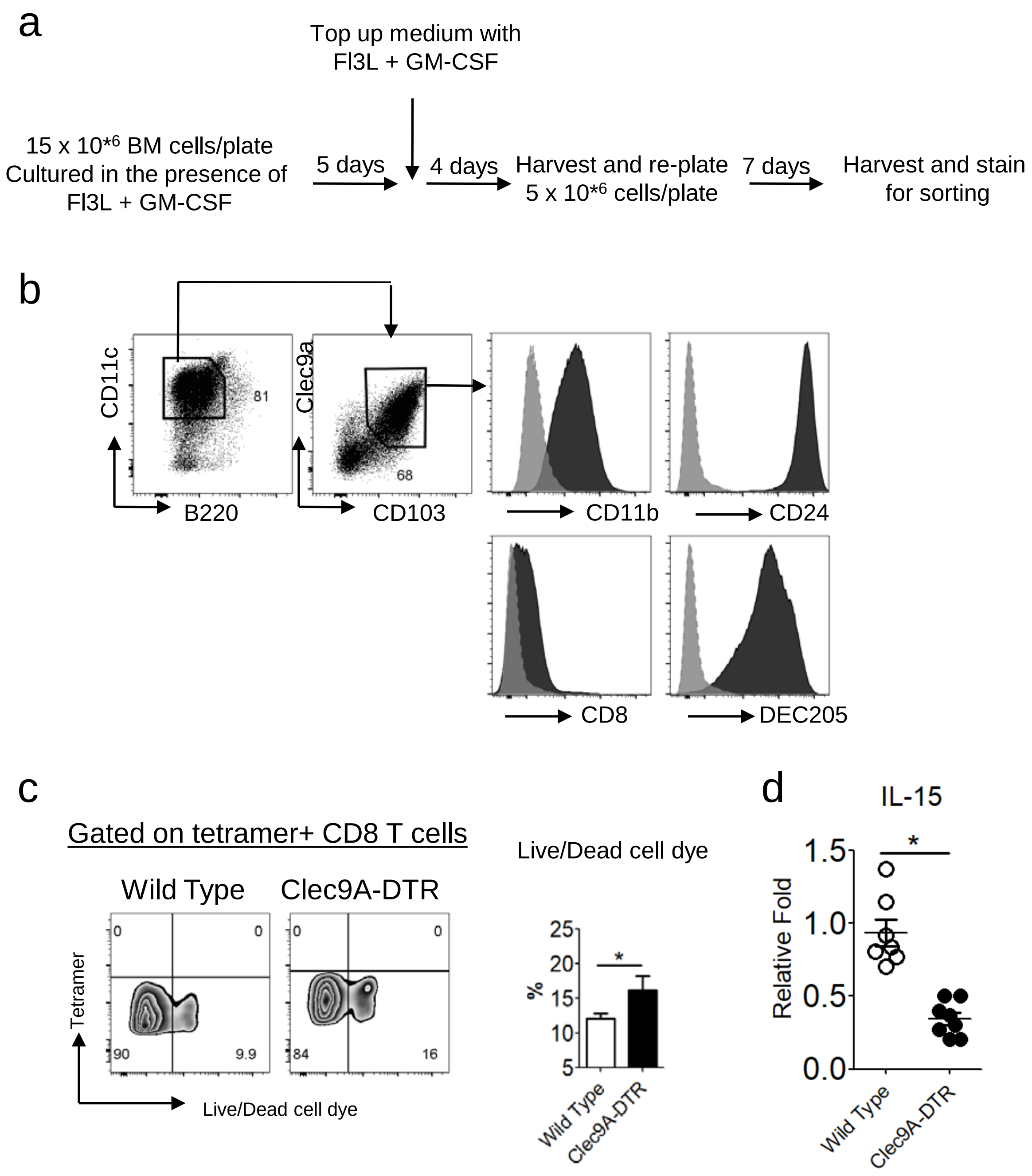

Supplementary Fig. 5: (a) Generation of inducible CD103^{+} cDCs (iCD103^{+} cDC) from bone marrow cells in a 16-day culture. (b) iCD103^{+} cDCs express CD103, Clec9a, CD11b, CD24, and DEC205. (c) Lung cells pre-gated on NP$_{366-374}$ - CD8^{+} T cells. Comparison of NP$_{366-374}$ - CD8^{+} T cells from the lungs of infected wild type and Clec9A-DTR mice (day 10 post infection) stained with fixable live/dead cell dye. (d) Relative transcript level for IL-15 in the lung of wild type and Clec9A-DTR mice on day 10 of infection as measured by quantitative real-time PCR. Data are shown as mean ± SEM. *, $p < 0.05$. Data represent two independent experiments (Total n = 7).

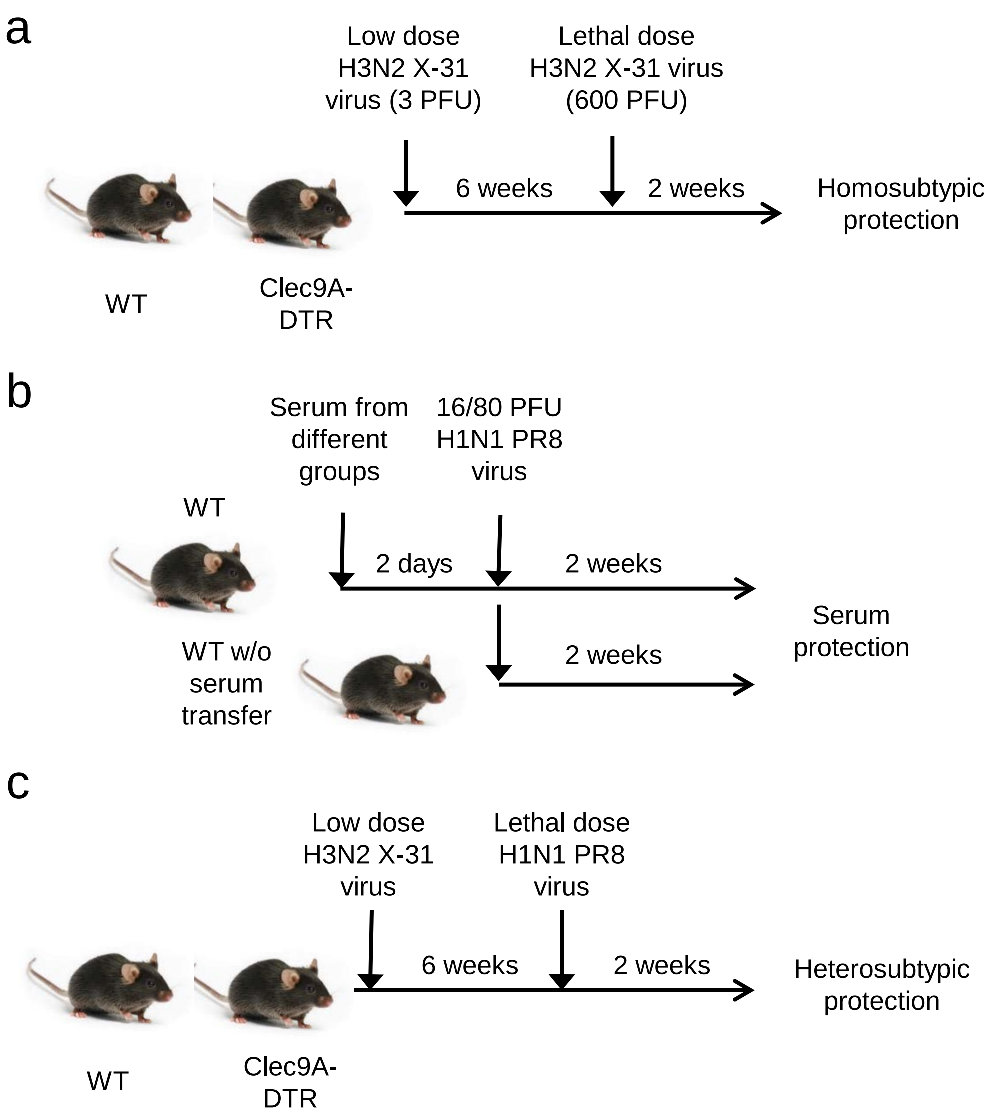

Supplementary Fig. 6: (a) For the homosubtypic experimental set-up, wild type and Clec9A-DTR mice were infected with 3 PFU of X-31 virus and housed for 6 weeks before secondary challenge with 600 PFU of X-31 virus. After secondary challenge, mice were monitored for at least 2 weeks. Negative control mice infected only with 600 PFU of X-31 virus were used as a positive control. (b) For the serum protection experimental set-up, sera were first harvested from uninfected mice, PR8-infected wild type, and PR8-infected Clec9A-DTR mice, and then transferred to naïve wild type mice infected 2 days later with 16/80 PFU of PR8 virus. Recipients were monitored for at least 2 weeks. Negative control mice not receiving serum were included as a positive control. (c) For the heterosubtypic experimental set-up, wild type and Clec9A-DTR mice were infected with 3 PFU of X-31 virus and housed for 6 weeks before secondary challenge with 600 PFU of PR8 virus. After secondary challenge, mice were monitored for at least 2 weeks. Negative control mice infected only with 600 PFU of PR8 virus were used as a positive control.
